# Modulation of Beta Oscillations in the Pallidum During Externally Cued Gait

**DOI:** 10.3389/frsip.2022.813509

**Published:** 2022-05-20

**Authors:** Chiahao Lu, Sommer L. Amundsen-Huffmaster, Kenneth H. Louie, Matthew N. Petrucci, Tara Palnitkar, Remi Patriat, Noam Harel, Michael C. Park, Jerrold L. Vitek, Colum D. MacKinnon, Scott E. Cooper

**Affiliations:** 1 Department of Neurology, University of Minnesota, Minneapolis, MN, United States; 2 Department of Biomedical Engineering, University of Minnesota, Minneapolis, MN, United States; 3 Department of Radiology, University of Minnesota, Minneapolis, MN, United States; 4 Department of Neurosurgery, University of Minnesota, Minneapolis, MN, United States

**Keywords:** Local field potential (LFP), Parkinson’s disease, external cueing, gait disorder, freezing of gait (FOG)

## Abstract

Freezing of gait (FOG) is a particularly debilitating symptom of Parkinson’s disease (PD) and is often refractory to treatment. A striking feature of FOG is that external sensory cues can be used to overcome freezing and improve gait. Local field potentials (LFPs) recorded from the subthalamic nucleus (STN) and globus pallidus (GP) show that beta-band power modulates with gait phase. In the STN, beta-band oscillations are modulated by external cues, but it is unknown if this relationship holds in the globus pallidus (GP). Here we report LFP data recorded from the left GP, using a Medtronic PC + S device, in a 68-year-old man with PD and FOG during treadmill walking. A “stepping stone” task was used during which stepping was cued using visual targets of constant color or targets that unpredictably changed color, requiring a step length adjustment. Gait performance was quantified using measures of treadmill ground reaction forces and center of pressure and body kinematics from video monitoring. Beta-band power (12–30 Hz) and number of freezing episodes were measured. Cues which unpredictably changed color improved FOG more than conventional cues and were associated with greater modulation of beta-band power in phase with gait. This preliminary finding suggests that cueing-induced improvement of FOG may relate to beta-band modulation.

## INTRODUCTION

Gait disorder is one of the most disabling motor symptoms of Parkinson’s disease (PD), particularly the phenomenon of freezing of gait (FOG). FOG is defined as a “brief, episodic absence or marked reduction of forward progression of the feet despite the intention to walk” (Giladi et al., 1992; Nutt et al., 2011) and is often difficult to treat (Bloem et al., 2004). Yet, one of the paradoxes of PD is that external sensory cues can markedly facilitate gait and reduce the occurrence and severity of FOG episodes (Nieuwboer, 2008). One proposed mechanism of cueing is by directing attentional resources to gait, in compensation for the loss of gait automaticity (Wu et al., 2015). However, the underlying mechanism by which external cues reduce FOG and improve gait is poorly understood.

Recent findings from studies that recorded local field potentials (LFPs) from implanted deep brain stimulation (DBS) leads showed that neural activity in the basal ganglia modulates with gait phase (Singh et al., 2011; Singh et al., 2013; Fischer et al., 2018; Hell et al., 2018). Moreover, it has been shown that external cueing is associated with an increase in beta-band modulation in the subthalamic nucleus (STN) during stepping (Fischer et al., 2018). Whether this relationship holds in the globus pallidus (GP) remains to be determined. In this study, we examined how two kinds of visual cueing strategies affected GP oscillations, freezing episodes and step lengths, during treadmill walking in a PD patient with severe FOG.

## MATERIALS AND METHODS

### Participant

A 68-year-old man who had been diagnosed with PD for 11 years was unilaterally implanted with a Medtronic 3389 electrode in the left GP and a Medtronic Activa PC + S sensing pulse generator (Afshar et al., 2013). His OFF-meds MDS-Unified Parkinson’s Disease Rating Scale (MDS-UPDRS) III motor score (Goetz et al., 2008) was 50 and freezing of gait questionnaire (Nieuwboer et al., 2009) score was 13. His levodopa daily equivalent dosage was 1,025 mg (Tomlinson et al., 2010). He gave informed consent and the protocol was approved by the University of Minnesota Institutional Review Board.

### Protocol

The participant was tested in the morning after 12-h withdrawal from antiparkinsonian medications (practically defined OFF-medication state) and DBS ON. He performed the walking task on an instrumented treadmill (0.7 m × 2.5 m; Model: C-Mill (van der Veen et al., 2018), Motekforce Link, Culemburg, Netherlands). The patient performed a “stepping stone” task during which visual cues (rectangles 0.22 m × 0.15 m) were projected via the built-in projector of the treadmill system onto the treadmill belt and moved with the belt at the corresponding belt speed. The rectangles were displaced left and right of the midline by 0.05 m, corresponding to left and right feet and spaced 0.11 m apart in the sagittal plane following initial titration to find settings which best alleviated the very severe gait impairment. Treadmill speed was set to 0.25 m/s on the same basis. Due to the severity of freezing, the patient was unable to walk on the treadmill without cues. Two visual cueing conditions were tested: stepping stones of constant color and stepping stones that changed color. For the constant color task, the rectangles were blue and the participant was instructed to step on the rectangles. On color-changing trials, the cues were the same except that randomly one in six rectangles changed color to red-and-white stripes. The participant was instructed that, when the rectangle changed color, he should adjust his step to avoid stepping on the striped rectangle. The timing of obstacle appearance relative to the previous ipsilateral toe-off ranged from −1.0 to 0.5 s which formed a bell-shaped distribution of the likelihood of success of obstacle avoidance (see our previous publication for more details on this task (Lu et al., 2019)). Color-change trials alternated with constant color trials in blocks of 3, starting with constant color trials. Eighteen trials were collected with nine trials for each cueing condition. Each walking trial lasted 2 min with 5 min of seated rest after each trial. One incomplete trial with constant color cues was not included in the LFP analysis due to severe freezing that necessitated stopping the treadmill.

The LFP signal was sampled at 422 Hz and recorded between contacts 1 and 3, which bracketed the active stimulation contact, 2- (case +). Stimulation was applied at an amplitude of 3.5 V, a frequency of 140 Hz and a pulse width of 60 μs. LFP data from the PC + S was synchronized with center of pressure (COP) data from the treadmill in the following manner. Briefly turning off/on DBS provided a synchronization signal that was present on the LFP channel and could be detected from a surface EMG sensor (Delsys Trigno, United States) on the participant’s neck. Onset and offset of treadmill movement were recorded with an external tachometer (SU-780J-1, Servo-Tek, United States) connected in synch to the same A/D converter (National Instruments NI-6225, sampling at 1,000 Hz) as the EMG sensor. The onset/offset of treadmill movement was also recorded in another tachometer internal to the treadmill. The signal from the internal tachometer and COP data, sampled at 500 Hz, were exported from the treadmill system. This procedure allowed us to synchronize LFPs with EMG, EMG with treadmill movement, and treadmill movement with COP, from which gait phase was determined as described below. Video of the lower extremities was recorded at 60 Hz with a spatial resolution of 720p (HDR-CX700, Sony, Tokyo, Japan).

### Electrode Localization

**FIGURE 1 ∣:**
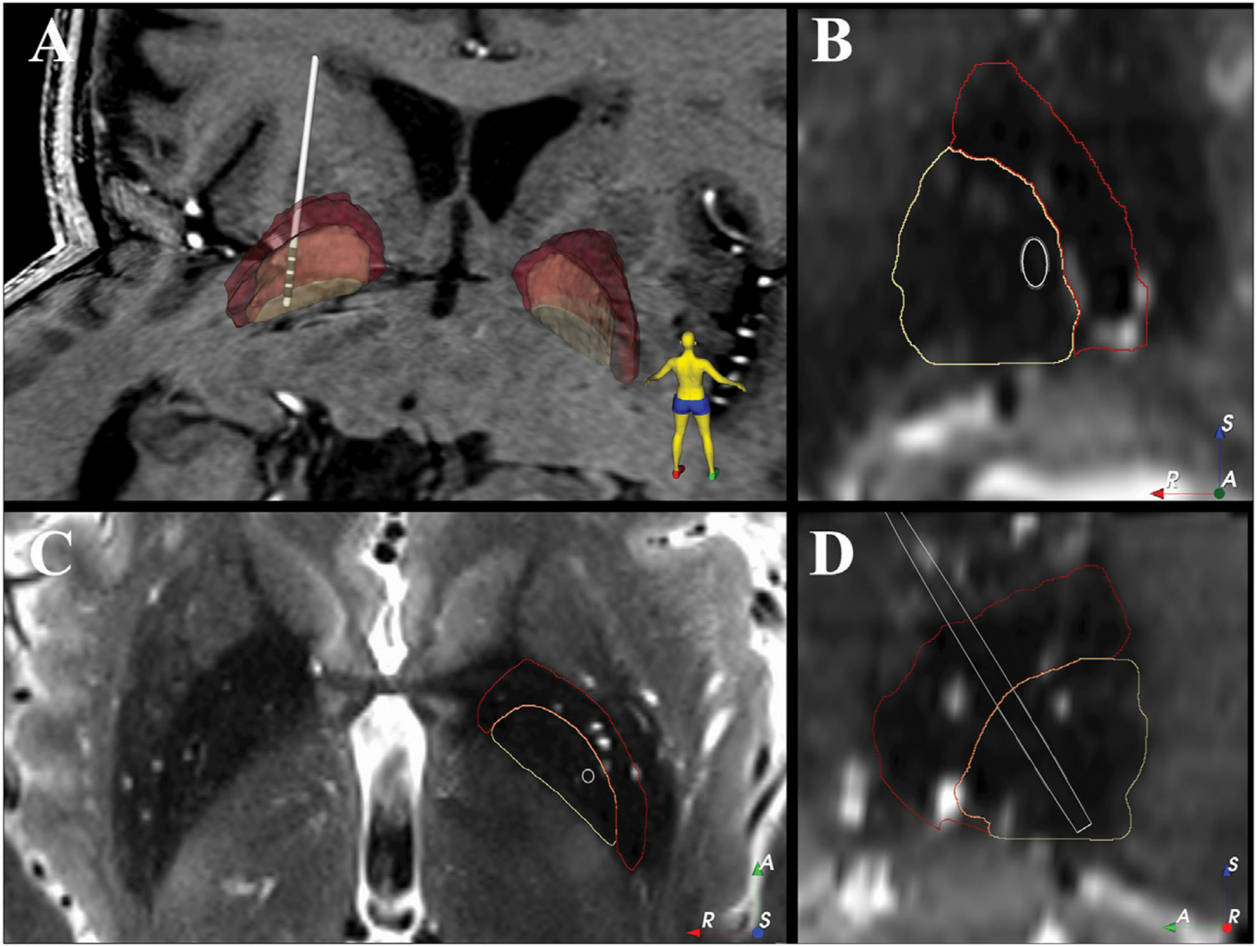
Electrode location within the patient’s GPi. **(A)** 3D view of the electrode location against the patient’s 7T T1 image. **(B–D)** show the coronal, axial and sagittal view of the electrode location against the patient’s 7T T2 image. The 2D images are shown in “left is right” orientation. White = electrode shaft, grey = electrode contact, beige = GPi, red = GPe, A = anterior, R = right, S = superior.

The patient was scanned preoperatively on a 7 T MRI scanner (Magnetom 7 T, Siemens Erlangen, Germany), equipped with SC72 gradients capable of 70 mT/m and a 200 T/m/s slew rate using a 32-element head array coil (Nova Medical, Inc. Burlington, MA, United States). Dielectric pads were utilized to enhance signal in the temporal regions (Teeuwisse et al., 2012). The scan protocol consisted of: T1-weighted whole brain scan (0.6 mm^3^ isotropic) and a high resolution T2-weighted axial slab covering from the top of the thalamus to the base of the substantia nigra with (0.4 × 0.4 × 1.0 mm^3^) voxel size. The globus pallidus internus (GPi) and globus pallidus externus (GPe) were manually segmented on the 7 T T2 axial image following out previous protocol (Duchin et al., 2018; Patriat et al., 2018). The GPi was bordered medially by the internal capsule, laterally and superiorly by the GPe (using the lateral medullary lamina as a border) and the ventral border of the GPi was identified by visual contrast to surrounding tissue and proximity to the optic tract. The segmentation was carried out using 3D Slicer (https://www.slicer.org/). The T2-weighted image was linearly co-registered to the patient’s T1-image using the Advanced Normalization Tools (Avants et al., 2011). Postoperative CT imaging was performed several weeks following the day of surgery. The postoperative CT was obtained with a Siemens Biograph64 Sensation with 0.6 mm slice thickness, kV 120, 512 × 512 matrix, FOV 260 mm^2^, 315 mAs, with 0.0° gantry tilt. Electrode and contact locations (Figure 1) were determined by registering the CT and the T1 images using non-linear methods (Elastix) (Klein et al., 2010).

### Data Analysis

The raw time domain LFP signal was downloaded from the PC + S device and processed offline in Python. Using custom Python code, the LFP data was band-pass filtered between 12 and 30 Hz, squared, moving average filtered (with window size of 0.1 s), and square-rooted to get the envelope of beta-band RMS (see Supplementary Figure S1 for an example).

**FIGURE 2 ∣:**
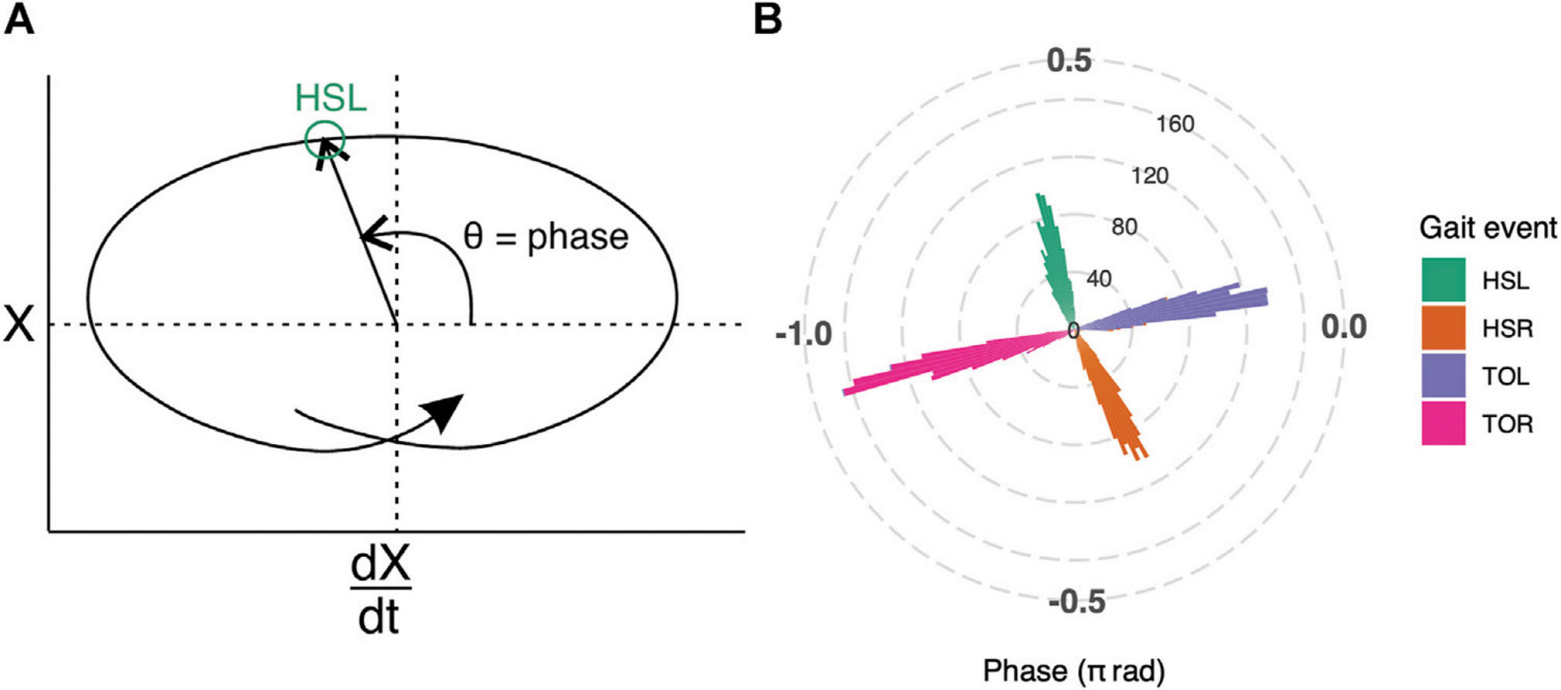
**(A)** Illustration of how gait phase was determined. Mediolateral COP displacement data are represented in the phase plane. The horizontal axis, dX/dt, is the first derivative of COPX with respect to time; the vertical axis, labeled as X, is the COP in the mediolateral direction. The origin is set at the average value over the entire trial. Polar coordinates (θ) derived from the phase plane, were used to determine the gait phase (see Supplementary Figure S2 for plot with real gait events). **(B)** Gait phase was validated against gait events that were independently detected by the treadmill manufacturer’s software for all trials. The phase of the gait events are represented as a polar histogram, where left heel-strike (HSL, green) occurs approximately at 0.59π radians, right toe-off (TOR, pink) at −0.90π radians, right heel-strike (HSR, orange) at −0.34π radians, and left toe-off (TOL, purple) at 0.06π radians.

The treadmill movement and COP trajectory were down-sampled and interpolated (scipy.signal.decimate, numpy.interp) (Virtanen et al., 2020; Harris et al., 2020) to match the LFP sampling frequency. COP X coordinate (mediolateral axis) was represented in the phase plane (X vs. dX/dt), in polar coordinates (r, theta) with origin set at the average for the whole trial (Figure 2A, Supplementary Figure S2). The theta value was extracted as the gait phase and this measure was validated against heel-strike and toe-off events independently detected by the treadmill manufacturer’s software (Figure 2B). A LOWESS smooth function (statsmodels.nonparametric.smoothers_lowess.lowess) (Seabold and Perktold, 2010) was applied to a plot of beta-band RMS vs. gait phase. To ensure continuity across phase “wrap-around” boundaries (i.e., −π radians is the same as +π radians), three copies of the data were spliced together, the LOWESS was fit, and then the first and last copies were discarded. Thus, the smoothing algorithm was applied to all strides within a trial.

A linear mixed effects model with 12–30 Hz beta power values as dependent variable was constructed to examine the effect of cueing on the LFP. Condition (color changing vs. non-color changing cues), Gait phase (stance vs. swing phase of the right leg, contralateral to the DBS implant), and their interaction (Condition by Gait phase) were fixed factors with Trial as the random-intercept term. In order to extract the beta power for each phase, we defined each phase using the average gait phase value (swing: −0.90π to −0.34π radians; stance: 0.06π to 0.59π radians) with events of bilateral heel-strikes and toe-offs exported from the instrumented treadmill. Then, we used the LFP sampling frequency and the average duration of a full gait cycle to estimate the number of data points in each phase. In each trial, average power value was computed for each gait phase; then these average power values were entered into the mixed model.

For analysis of FOG occurrences, number of freezing episodes were logged from the video record by an experienced, fellowship-trained movement disorders neurologist (S.C.). Since the count was non-normally distributed (Shapiro-Walk *W* = 0.85, *p* < 0.01), a non-parametric Wilcoxon signed rank paired sample test was used to examine the difference in freezing episodes between the trials with vs. without color-changing cues. Step length was computed from heel-strike and toe-off events exported by the treadmill software, which estimated them from the COP trajectory, independently of the COP gait phase analysis. These gait events were also used to validate the COP-determined gait phase. Step lengths were normally distributed (*W* = 0.97, *p* = 0.72) so a paired *t*-test was used to examine the differences in step length between the two cueing conditions, each side analyzed separately, with Bonferroni correction. The significance level was set at 0.05. All statistical analyses were performed in R (R Core Team, 2018).

## RESULTS

Reconstruction of the electrode location shows that the most dorsal contact (contact 3) was near the border of GPi and GPe with the other contacts (contacts 0–2) located within GPi (Figure 1).

The gait phase estimation based on COP data was reliable; heel-strike and toe-off events, as independently detected by the built-in treadmill software, occurred at consistent gait phases (Figure 2B). The average phase values for left heel-strike (HSL) were 0.59π radians (95% CI: 0.59π to 0.60π radians), right heel-strike (HSR) were −0.34π radians (95% CI: −0.35π to −0.34π radians), left toe-off (TOL) were 0.06π radians (95% CI: 0.06π to 0.07π radians) and right toe-off (TOR) were −0.90π radians (95% CI: −0.91π to −0.90π radians).

**FIGURE 3 ∣:**
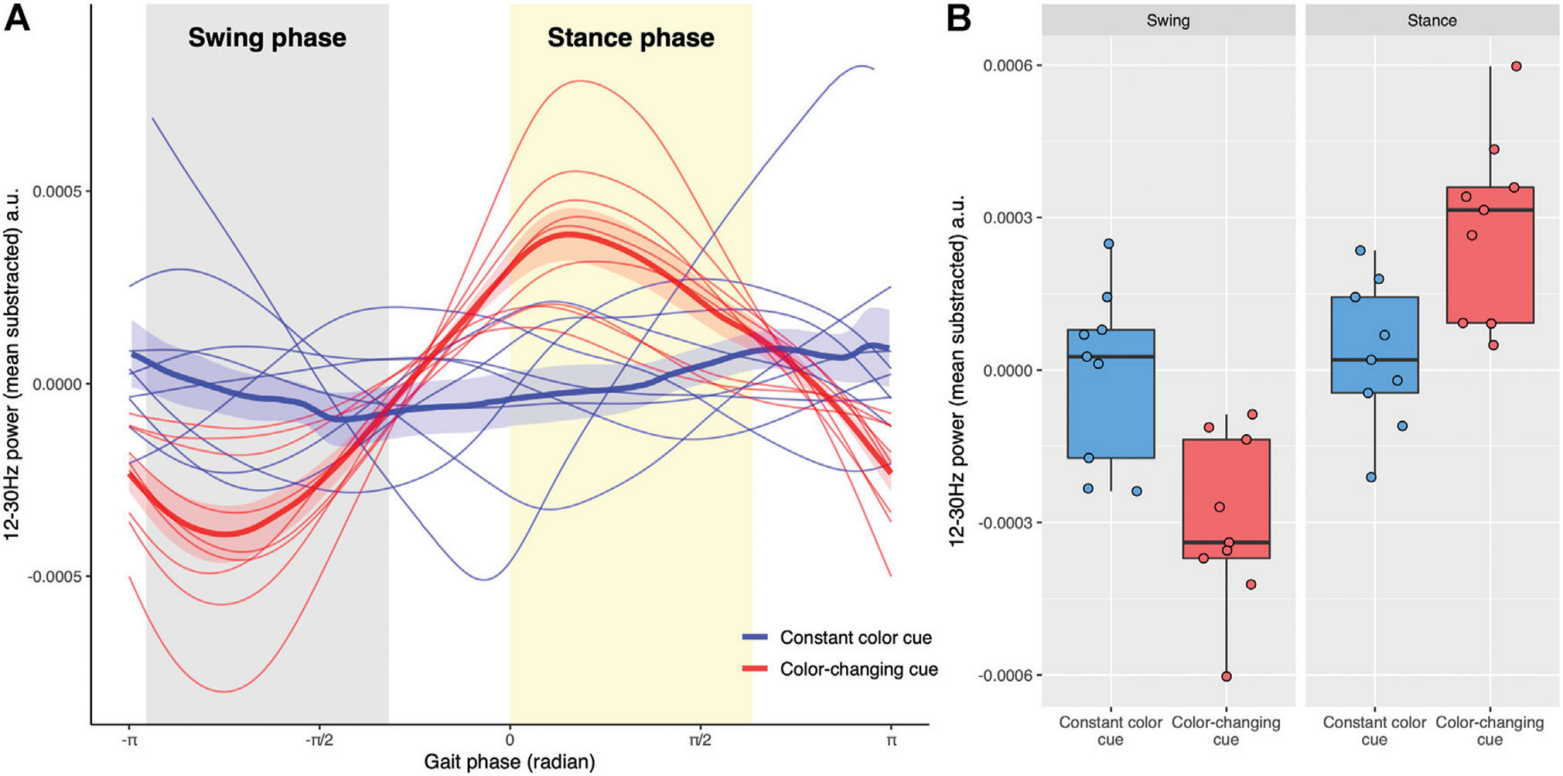
**(A)** The relationship between total beta-power (12–30 Hz) and gait phases. The *swing phase* of the right leg (gray shaded area) is from −0.90π to −0.34π radians while the stance phase (yellow shaded area) of the right leg is between 0 and 0.60π radians. The gait phase was determined including all strides in each trial. Trials with constant color cues are blue, whereas trials with color-changing cues are red. The thick lines represent the average power across trials and the associated shaded region shows the standard error of the mean across trials. **(B)** Boxplots show total beta-power in swing and stance phases for two types of visual cues. Each point represents the average power of each trial in each phase. Trials with constant color cues are blue whereas trials with color-changing cues are red. The Condition by Gait phase interaction for the total beta power was significant (*p* < 0.001), reflecting that the power of the color-changing cues was lower in the swing phase and higher in the stance phase compared to non-color changing cues.

Figure 3A demonstrates that beta frequency power modulated more deeply with gait phase on trials with color-changing cues than on trials with a constant color cue (see Supplementary Figure S3 for spectrograms). The mixed model analysis showed significant Gait phase (*F*_(1,32)_ = 31.15, *p* < 0.001) and Condition by Gait phase interaction (*F*_(1, 32)_ = 24.30, *p* < 0.001) effects. The significant interaction effect reflected that the differences between color-changing cues and constant color cues were different between swing phase and stance phase (Figure 3B). Post-hoc *t*-tests showed that the 12–30 Hz power in color changing cues was lower during swing phase (*t*_(32)_ = 3.73, *p* < 0.001) and greater during stance phase (*t*_(32)_ = −3.24, *p* < 0.01) compared to constant color cues. The Condition was not statistically significant (*F*_(1, 32)_ = 0.12, *p* = 0.73).

**FIGURE 4 ∣:**
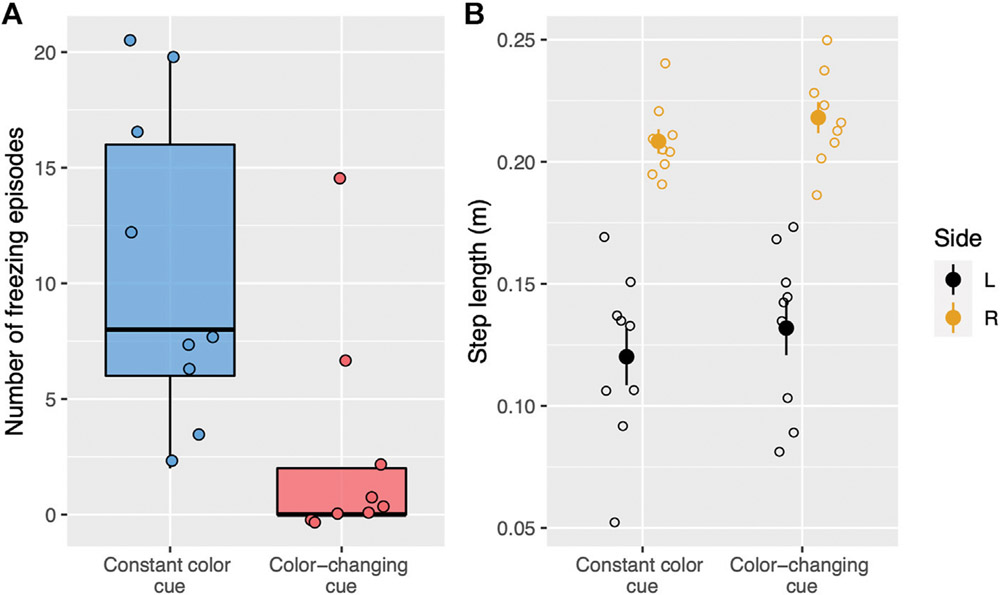
**(A)** Boxplots show the number of freezing episodes during treadmill walking for two types of visual cues. Each dot represents a single trial. Trials with constant color cues are blue whereas trials with color-changing cues (stepping stones change color) are red. **(B)** Step length for each side and visual cue condition (constant color, color-changing) during treadmill walking. Each open circle represents the average step length for a single trial. The filled circle represents the average step length for each cueing condition and each side and the vertical lines represent standard error. The right side is yellow whereas the left side is black.

During treadmill walking, there were significantly fewer freezing episodes (*W* = 72, *p* < 0.01, Figure 4A) on trials with color-changing-cues (median: 0, IQR: 2) compared to constant color cues (median:8, IQR:10). The average step lengths for both sides were similar in the color-changing (Figure 4B, mean ± SD, right: 0.22 ± 0.02 m; left: 0.13 ± 0.03 m) and the constant color (right: 0.21 ± 0.01 m; left: 0.12 ± 0.04 m) cueing conditions. The statistical tests showed no significant difference between the two cueing types on the average step length for each side (right: *t*_(8)_ = −0.92, *p* = 0.76; left: *t*_(8)_ = −0.60, *p* = 1.00).

## DISCUSSION

In this study, we recorded LFPs in GP during treadmill walking with two types of visual cueing. The results showed that: 1) the magnitude of beta-band oscillations modulated with phase of gait, 2) visual cues reduced freezing and facilitated gait (the participant was unable to walk without cues) but color-changing cues, requiring a fast adjustment of stepping, improved FOG more than constant color cues, 3) beta-band modulation was greater with the color-changing cues compared to constant color cues.

Previous studies with STN LFP recordings have shown beta modulation with gait phase (Singh et al., 2013; Fischer et al., 2018; Hell et al., 2018), but this was not seen for pallidal LFPs in the single published study (Singh et al., 2011). This discrepancy might be because the patients in the single GP study had dystonia and not PD with FOG. Another explanation may be that they were studied in the acute post-DBS-implant where microlesion effects may affect the underlying neural dynamics (Mann et al., 2009; Mestre et al., 2016), rather than in the chronically implanted state. Our findings are similar to the results reported from the STN in PD patients. Fischer et al. (2018) demonstrated that beta-band power during stepping increased with auditory cueing. Hell et al. (2018) further showed that beta-band power in the STN is modulated with gait phase. They also found that STN-DBS improved gait but that improvements in gait metrics were not accompanied by an attenuation of high beta power. Our results suggest that increased depth of beta modulation, rather than overall beta attenuation, is associated with improvements in FOG. Suppression of synchronized beta oscillations is believed to be the means by which DBS improves symptoms in PD (Afshar et al., 2013), but the response of gait symptoms to DBS is less consistent and less persistent than of other PD symptoms (St. George et al., 2010). This may be because conventional (isochronous, constant, unvarying) DBS suppresses beta throughout the gait cycle, whereas intermittent DBS, if synchronized with the gait cycle, might reinforce the “troughs” of naturally occurring gait phase modulation, increasing depth of modulation. Recent advancements in real-time DBS systems that can selectively facilitate or suppress beta-band activity (Petrucci et al., 2020; Escobar Sanabria et al., 2021) might provide the means to deliver closed-loop DBS system that modulates beta-band activity in relation to timing of gait events in order to improve freezing in individuals with PD.

Why did unpredictable changes in the color of the stepping stone, which required adjustment of the ongoing gait cycle, improve FOG more than ordinary cues? It has been proposed that gait disturbances arise, in part, from a loss of automaticity and an increased reliance on attention to maintain the gait cycle (Wu et al., 2015; Ginis et al., 2018). The changing color cueing condition may direct more attention toward gait, thereby improving compensation for the loss of automaticity (Wu et al., 2015; Ginis et al., 2018). Despite the marked change in FOG, the difference in step length between the two visual cues was nonsignificant. This is not surprising because the participant was instructed to step on the projected blue rectangles which restricted any variation in step length.

It is possible that the difference in modulation of beta-band power between the two cueing types might be a result, rather than the cause, of the decreased festination (i.e., shortening of steps progressively) which is strongly related to freezing (Iansek et al., 2006). We cannot exclude this possibility, but it is unlikely because step length did not differ significantly between the two cueing conditions. A strength of the present study is that we were able to obtain a large quantity of gait data in a patient with very severe freezing. However, our results can only be considered preliminary, as this is a single-patient case report. A further limitation is that we were unable to obtain LFP recordings during uncued gait and/or during off-stimulation state due to the participant’s inability to walk under either of those conditions. Future studies may consider include individuals with a variety of disease severity in order to understand how cueing affects pallidal activity during walking.

## Supplementary Material

Supplementary material

## Data Availability

The raw data supporting the conclusion of this article will be made available by the authors, without undue reservation.
