# Supplementary material for: Modulation of Beta Oscillations in the Pallidum During Externally Cued Gait
Source: Front Signal Process (Lausanne). Author manuscript; Available in PMC 2022 Jun 4. (PMC9164277; doi:10.3389/frsip.2022.813509)
Supplement: Supplementary material [file NIHMS1810278-supplement-Supplementary_material.pdf]

Supplementary Material

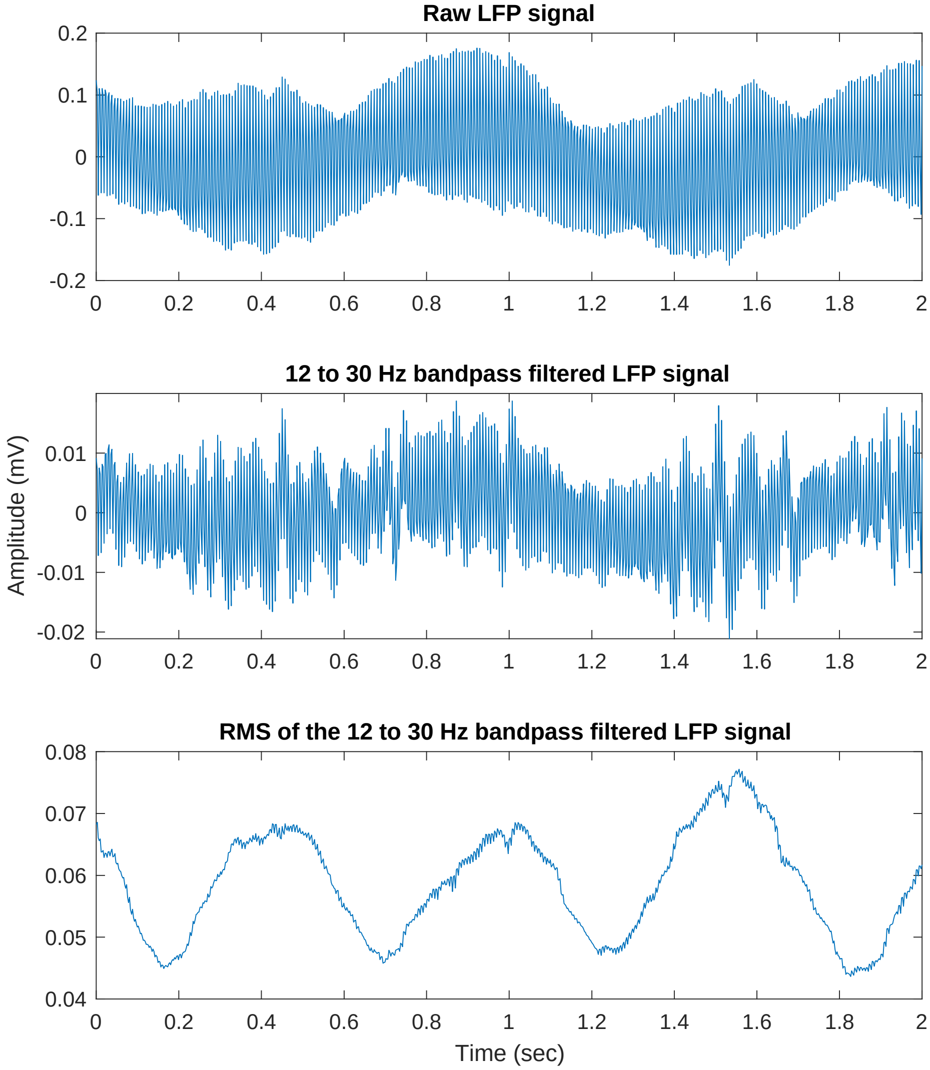

Figure S1. Example of a 2 second time series data from the recorded local field potential (LFP) signal. The top row shows the raw LFP signal; the middle row shows the 12 to 30 Hz bandpass filtered signal and the bottom row shows the root mean square (RMS) of the bandpass filtered signal, i.e. linear envelope of the bandpass filtered LFP.

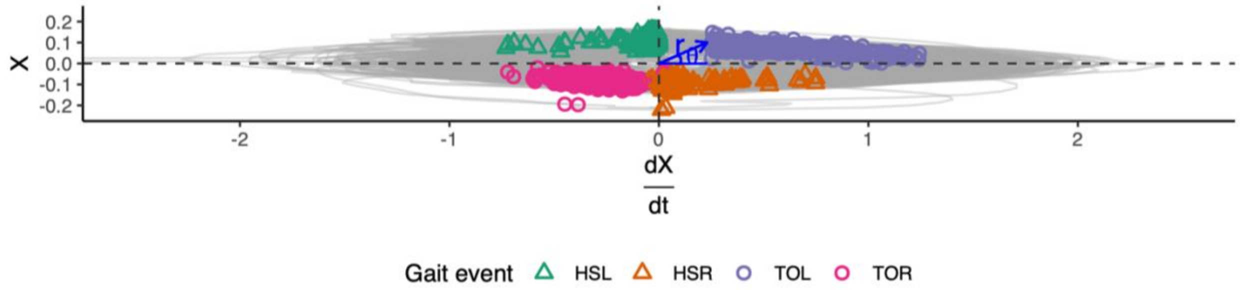

Figure S2, Gait phase estimation with all gait trials. The horizontal axis, dX/dt, is the first derivative of COPX with respect to time; the vertical axis, labeled as X, is the COP in the mediolateral direction. Mediolateral COP displacement data are represented in the phase plane as gray traces. Gait events detected from the treadmill software are shown as triangles representing heel strikes (green for left heel-strikes, HSL; orange for right, HSR) and circles representing toe-offs (purple for left, TOL; pink for right, TOR). The origin was set for each trial at the average value of dX/dt and COPX over that entire trial. Polar coordinate (θ) was derived from the phase plane as the gait phase. Note that X and dX/dt have different units, which is why the plot appears "flattened" on the vertical axis.

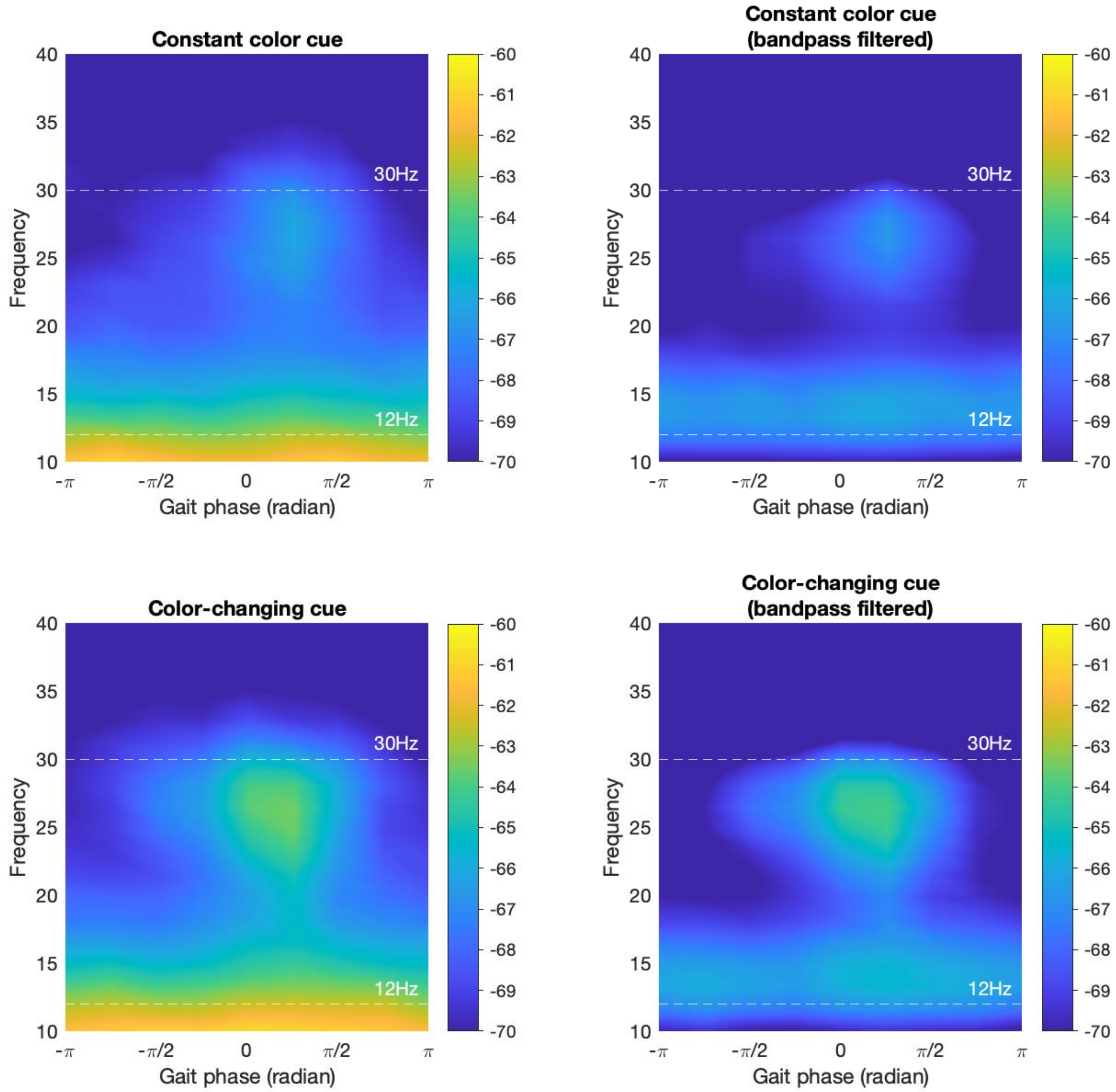

Figure S3. The average spectrogram for each cueing condition (left column: raw LFP signal; right column: 12 to 30 Hz bandpass filtered LFP signal). The top row plots show the average spectrogram for constant color cueing and the bottom row plots are for the color-changing cueing condition. The x-axis is set to the gait phase computed with center of pressure data. The white dashed lines denote 12 and 30 Hz frequencies.
